# Quercetin alleviates liver fibrosis via regulating glycolysis of liver sinusoidal endothelial cells and neutrophil infiltration

**DOI:** 10.17305/bb.2024.10530

**Published:** 2024-12-01

**Authors:** Xiaoying Chen, Yifan Wang, Jie Wan, Xiaoyun Dou, Chuzhao Zhang, Meng Sun, Fang Ye

**Affiliations:** 1 The First Clinical Medical College, Nanjing University of Chinese Medicine, Nanjing, Jiangsu, China; 2 School of Traditional Chinese Medicine, Nanjing University of Chinese Medicine, Nanjing, Jiangsu, China; 3 Department of Plastic Surgery and Burn Center, Second Affiliated Hospital, Shantou University Medical College, Shantou, Guangdong, China

**Keywords:** Quercetin (QE), liver fibrosis, glycolysis, liver sinusoidal endothelial cell (LSEC), neutrophil infiltration

## Abstract

Liver fibrosis, a common characteristic in various chronic liver diseases, is largely influenced by glycolysis. Quercetin (QE), a natural flavonoid known to regulate glycolysis, was studied for its effects on liver fibrosis and its underlying mechanism. In a model of liver fibrosis induced by carbon tetrachloride (CCl4), we aimed to assess pathological features, serum marker levels, and analyze the expression of glycolysis-related enzymes at both mRNA and protein levels, with a focus on changes in liver sinusoidal endothelial cells (LSECs). Our results showed that QE effectively improved liver injury and fibrosis evident by improved pathological features and lowered levels of serum markers, such as alanine aminotransferase (ALT), aspartate aminotransferase (AST), alkaline phosphatase (ALP), γ-glutamyl transferase (GGT), total bile acid (TBA), total bilirubin (TBIL), direct bilirubin (DBIL), hyaluronic acid (HA), laminin (LN), and procollagen type III (PCIII). QE also decreased lactate production and downregulated the expression of glycolysis-related enzymes—pyruvate kinase M2 (PKM2), phosphofructokinase platelet (PFKP), and hexokinase II (HK2)—at both the mRNA and protein levels. QE reduced the expression and activity of these enzymes, resulting in reduced glucose consumption, adenosine triphosphate (ATP) production, and lactate generation. Further analysis revealed that QE inhibited the production of chemokine (C-X-C motif) ligand 1 (CXCL1) and suppressed neutrophil recruitment. Overall, QE showed promising therapeutic potential for liver fibrosis by targeting LSEC glycolysis and reducing neutrophil infiltration.

## Introduction

Liver fibrosis is a dynamic pathological progression of various chronic liver diseases (CLDs) [1, 2]. Without intervention, it can advance to cirrhosis, liver cancer, and even liver failure [3, 4]. Considering that current drugs for liver fibrosis treatment are limited and ineffective, the development of anti-fibrotic medications is an urgent issue [5].

The liver can maintain metabolic homeostasis, but various unfavorable conditions can impair its function, especially the abnormal activation of glucose metabolic pathways, such as glycolysis, which accelerates the progression of liver fibrosis [6]. Liver sinusoidal endothelial cells (LSECs) are a specific population of endothelial cells (ECs) that regulate liver function via angiocrine signaling [7, 8]. Chemokines may be essential to this signaling program by attracting neutrophils and thrombocytes, promoting inflammation and sinus thrombosis, and increasing portal vein pressure [9, 10]. Recent studies have indicated the potential of heightened LSEC-specific glycolysis to increase chemokine (C-X-C motif) ligand 1 (CXCL1) expression [11]. Conversely, inhibition of glycolytic processes in LSECs impedes the production of CXCL1 and neutrophil infiltration, thus ameliorating the progression of liver fibrosis [12]. These findings highlight the importance of targeting LSEC glycolysis as a main treatment strategy for liver fibrosis.

Quercetin (QE), a naturally occurring bioactive flavonoid [13], has demonstrated multiple pharmacological activities in preclinical trials, including anti-inflammatory, antioxidant, and neuroprotective effects [14–16]. Recent studies have shown that QE has potent anti-fibrotic properties, providing new possibilities for the treatment of fibrosis-related diseases [17, 18]. In addition, the role of QE in glycolysis has attracted the attention of researchers. QE inhibits glycolysis in oral squamous cell carcinoma via the G3BP1/YWHAZ axis [19]. It also antagonizes glucose fluctuation-induced renal injury by inhibiting aerobic glycolysis [20]. However, the effect of QE on glycolysis during liver fibrosis and its potential to regulate this metabolic pathway in LSECs remain unexplored. Elucidation of these mechanisms could provide valuable insights into the anti-fibrotic effects of QE and provide novel therapeutic targets for treating liver fibrosis.

## Materials and methods

### Animals and model establishment

Male C57BL/6 mice (20–24 g) were purchased from the Zhejiang Academy of Medical Sciences (authorization number: SCXK [Zhe] 2019-0004). All animals were raised at Nanjing University of Chinese Medicine (Nanjing, China) following the institutional and local committee approval for experimental procedures. Animal welfare was maintained according to the National Institutes of Health guidelines. A total of 40 mice were randomly divided into five groups (*n* ═ 8 in each group), including the control group, model (CCl4-treated) group, low dose of QE co-treated (L-QE) group, high dose of QE co-treated (H-QE) group, and colchicine co-treated (Col) group. The control group received injections of olive oil, while the other groups were injected intraperitoneally with 10% CCl4 (0.5 mL/kg body weight) diluted in olive oil twice a week for eight weeks [21, 22]. QE (purity ≥ 98.0%) was sourced from Sigma-Aldrich (St. Louis, MO, USA) and a 100 mg/mL stock was prepared using dimethylsulfoxide (DMSO) (Sigma-Aldrich, St. Louis, MO, USA). The stock was then diluted with phosphate-buffered saline (PBS) to obtain a 10 mL fine suspension. Starting from the fifth week, the L-QE and H-QE groups were orally administered corresponding doses of QE (25 and 50 mg/kg, respectively) [23]. The control and model groups were treated with the same volume of normal saline. Colchicine (dissolved in sterile PBS) at 0.2 mg/kg was orally administered to the Col group as the positive control [24]. At the end of the experiment, blood was sampled from the retro-orbital plexus under 3% isoflurane anesthesia, liver tissue was collected, and all mice were euthanized.

### The liver index

At the end of the experiment, body and liver weights were recorded. The liver index was calculated using the following formula: index (%) ═ (tissue weight (g) / body weight (g)) × 100% [25].

### Biochemical analysis of liver function

Serum samples were centrifuged at 3500 rpm for 10 min at 4 ^∘^C. AST, ALT, alkaline phosphatase (ALP), GGT, total bile acid (TBA), total bilirubin (TBIL), and direct bilirubin (DBIL) levels were determined using an automatic biochemical analyzer (Hitachi, Tokyo, Japan) to assess the degree of liver injury in each group.

### Detection of morphological changes

The liver tissues were fixed in 4% paraformaldehyde (PFA) and embedded in paraffin. Thin sections (4 µm) were prepared and stained. Hematoxylin and eosin (H&E) staining was used to assess histopathology. Masson and Sirius Red stains were used to examine collagen and muscle fiber alterations, respectively. A light microscope (Leica Microsystems, Wetzlar, Germany) was used to capture photographs of the stained sections in random fields.

### Detection of liver fibrosis markers

To evaluate fibrosis markers, the serum levels of hyaluronic acid (HA), laminin (LN), and procollagen type III (PCIII) were assessed using an enzyme-linked immunosorbent assay (ELISA) kit following the manufacturer’s guidelines. The ELISA kits for HA (MM-0514M1), LN (MM-0161M1), and PCIII (MM-0810M1) were from Jiangsu Meimian Industrial Co., Ltd. (Yancheng, Jiangsu, China).

### Western blotting

The protein concentration was determined by using the bicinchoninic acid (BCA) Protein Assay Kit (P0012, Beyotime Biotechnology, Shanghai, China) after extracting total proteins from LSECs and mouse liver tissues using a radio-immunoprecipitation assay (RIPA) lysis buffer. The proteins were then transferred to nitrocellulose membranes after undergoing sodium dodecyl sulfate-polyacrylamide gel electrophoresis. These membranes were blocked with 5% bovine serum albumin (BSA) and incubated with diluted primary antibodies, such as rabbit glyceraldehyde-3-phosphate dehydrogenase (GAPDH) (10494-1-AP, 1:5000, Proteintech Group), hexokinase II (22029-1-AP, 1:500, Proteintech Group), phosphofructokinase platelet (PFKP) (13389-1-AP, 1:2000, Proteintech Group), and pyruvate kinase M2 (PKM2) (15822-1-AP, 1:1000, Proteintech Group), overnight at 4 ^∘^C. The membranes were then washed three times with Tris-buffered saline with Tween (TBST) and incubated with secondary horseradish peroxidase (HRP)-labeled goat anti-rabbit IgG (ab205718, 1:2000, Abcam) for 1 h. After three additional washes, the blots were visualized using a chemiluminescence imager (Quantity One; Bio-Rad Laboratories Inc., Hercules, CA, USA) with GAPDH used as a control.

### Total RNA extraction and reverse transcription-quantitative polymerase chain reaction (RT-qPCR)

We extracted total RNA from LSECs or mouse liver tissues using TRIzol reagent. Next, we reverse-transcribed 2 µg of RNA into complementary DNA (cDNA) using a 20-µL reaction system and a reverse transcription kit (11123ES10, Yeasen Biotech Co., Ltd.). For quantitative real-time PCR (RT-qPCR), we used 1 µL of cDNA and SYBR Green Master Mix (11184ES08, Yeasen Biotech Co., Ltd.) to quantify the expression of relevant genes. We used beta-actin (β-actin) as the invariant control in each analysis. We conducted at least three repeated experiments. The RT-qPCR reaction conditions involved 40 cycles of 95 ^∘^C for 20 s, 54 ^∘^C for 30 s, and 72 ^∘^C for 30 s. We calculated the relative gene expression using the 2^−ΔΔCt^ method [26]. We obtained all primers from Bioengineering Co., Ltd. (Shanghai, China) and their sequences are listed in Table 1.

**Table 1:** The sequences of primers

**Gene name**	**Forward sequence**	**Reverse sequence**
Mouse-β-actin	GATGGTGGGAATGGGTCAGAAGG	TTGTAGAAGGTGTGGTGCCAGATC
Mouse-HK2	ATCAAAGAGAACAAGGGCGAGGAG	GCGGAGGAAGCGGACATCAC
Mouse-PFKP	CTGGCAAGGGAAGACAAGAACAAC	GAGCCGCACAGGACCGTATG
Mouse-PKM2	TGTGCCGCCTGGACATTGAC	AATTCAGCCGAGCCACATTCATTC

HK2: Hexokinase II; PFKP: Phosphofructokinase platelet; PKM2: Pyruvate kinase M2.

### Immunofluorescence (IF)

Thin sections (4 µm) of liver tissue were de-paraffinized using 5% BSA. Subsequently, they were incubated with corresponding primary antibody rabbit myeloperoxidase (MPO) (22225-1-AP, 1:50, Proteintech Group) at 4 ^∘^C overnight. After washing with PBS, they were incubated with the secondary antibody fluorescein-conjugated goat anti-rabbit IgG (H+L) (ZF-0311, 1:100, Zsgb-Bio) in the dark at 4 ^∘^C for 1 h. Nuclei were stained with 4′,6-diamidino-2-phenylindole (DAPI) (C1006, Beyotime Biotechnology, Jiangsu, China). Images were acquired using an inverted fluorescence microscope (Leica Microsystems). The images were taken blindly from randomly selected fields of view and changes in fluorescence were monitored.

### Immunohistochemistry (IHC)

Liver tissue sections were treated to remove wax and then exposed to 0.3% hydrogen peroxide to prevent natural peroxidase activity. They were then blocked with 5% BSA at room temperature for 30 min. Following a rinse with PBS, the sections were incubated at 4 ^∘^C overnight with a primary antibody (rabbit CXCL1, 12335-1-AP, 1:50, Proteintech Group). On the next day, the sections were washed again and exposed to an HRP-conjugated secondary antibody for 50 min. The chromogen diaminobenzidine (DAB) was used to produce color, and hematoxylin was applied for counterstaining. Finally, the prepared and stained sections were observed and photographed using a microscope (Leica Microsystems).

### Cell culture

Immortalized human LSECs (IM-H399) were purchased from IMMOCELL (Xiamen, Fujian, China). The cells were cultured in endothelial cell complete medium supplemented with 10% fetal bovine serum (FBS) and 100 U/mL of streptomycin and penicillin and incubated at 37 ^∘^C and 5% CO_2_. Vascular endothelial growth factor (VEGF) (Chamot Biotechnology, Shanghai, China) was dissolved in PBS at 40 ng/mL to stimulate the cells [27, 28]. QE was dissolved in DMSO and added to the medium with final concentrations of 0, 2, 4, 8, 16, and 32 µM for subsequent experiments. The final concentration of DMSO in the media was below 0.1%.

### Cell counting kit 8 (CCK8) assays

Cell viability was assessed using the CCK8 kit (C0038, Beyotime Biotechnology, Shanghai, China). The LSECs were seeded in 96-well plates and cultured overnight at 37 ^∘^C. After 24 h of cell treatment with different concentrations of QE, 10 µL of CCK8 reagent was introduced to each well and incubated for 2 h. Cell viability was determined by measuring the optical density (OD) at 450 nm.

### Measurement of lactate levels

Lactate levels in LSECs and mouse liver tissue were assessed using kits according to the manufacturer’s instructions (A019-2-1, Nanjing Jiancheng Bioengineering Institute, Nanjing, China).

### Glucose consumption assays

To assess glucose consumption in LSECs, a glucose oxidase (GOD)-based ELISA kit (ml024803, Meilian Biology Technology Co., Ltd., Shanghai, China) was employed. GOD catalyzes glucose oxidation to gluconic acid, providing an alternative indicator of glucose consumption [29]. Following the manufacturer’s protocols, samples, standards, and HRP-labeled antibodies were added to microwells coated with GOD antibodies. After incubation and washing, a TMB substrate was used to develop the color. The OD value was measured at 450 nm using a microplate reader to calculate the sample concentration.

### Measurement of adenosine triphosphate (ATP) levels

ATP levels in LSECs were determined using a luciferin–luciferase assay with an ATP Assay Kit (S0026, Beyotime Biotechnology, Shanghai, China) according to the manufacturer’s protocols.

### Glycolysis enzyme activity assays

The intracellular activities of HK2 (ml058727), PFKP (ml037256), and PKM2 (ml237485) in LSECs were measured using ELISA kits (Meilian Biology Technology Co., Ltd., Shanghai, China) according to the manufacturer’s protocols.

### MPO and neutrophil elastase (NE) activity assays

MPO and NE activity in liver tissue were measured using specific assay kits. MPO activity was determined using the colorimetric method with the MPO assay kit (A044-1-1, Nanjing Jiancheng Bioengineering Institute, Nanjing), while NE activity was measured using an ELISA kit (MM-0603M2, Meilian Biology Technology Co., Ltd., Shanghai, China). Both assays were performed following the respective manufacturer’s instructions.

### Measurement of CXCL1 levels

The CXCL1 level released in the culture medium was determined using an ELISA kit (ml057794, Meilian Biology Technology Co, Ltd., Shanghai, China) according to the manufacturer’s protocol.

**Figure 1.**
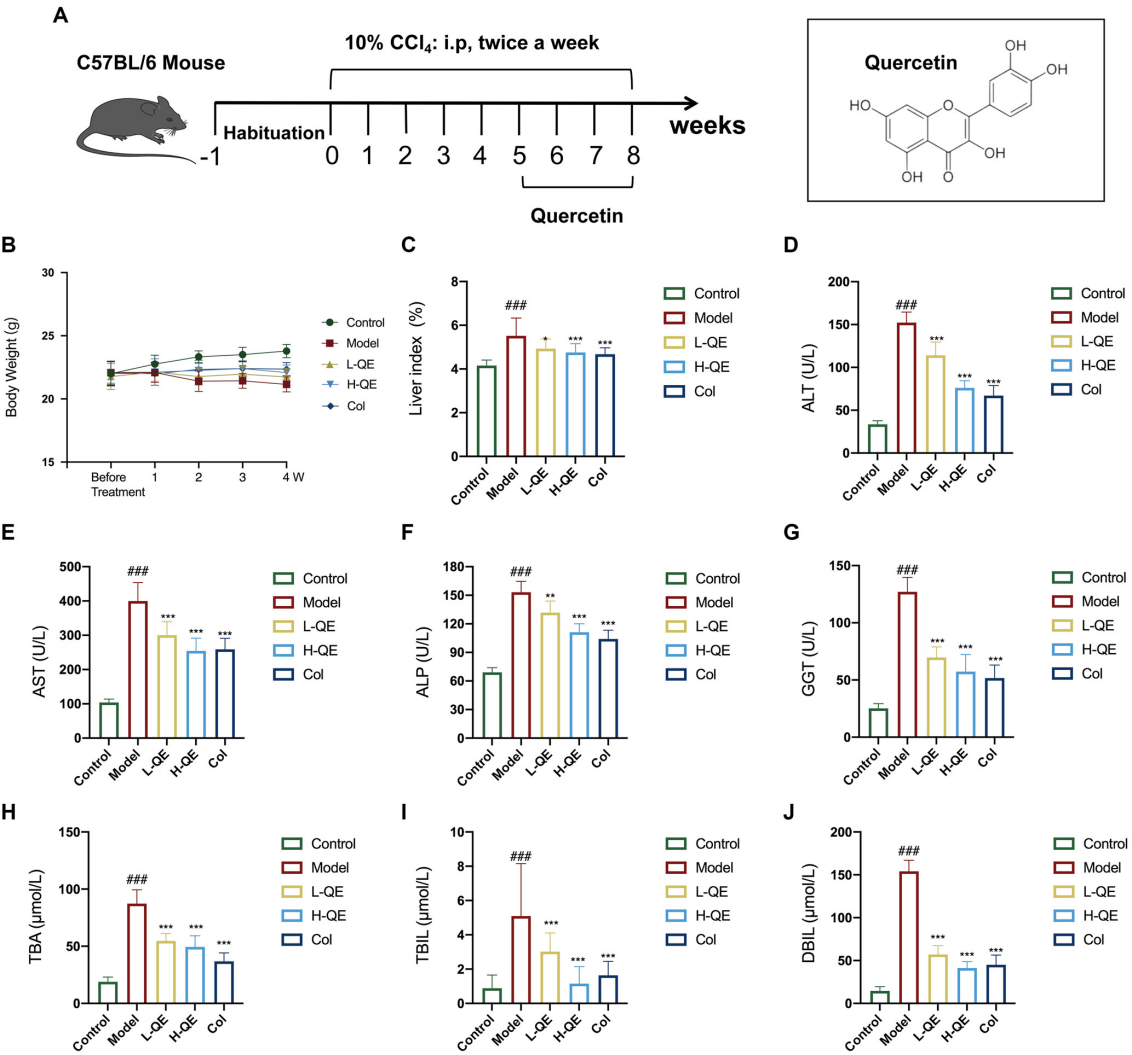
**Efficacy of QE in the treatment of liver injury**. (A) Diagram of QE treatment in a CCl4-induced liver fibrosis mouse model; (B) Body weight; (C) Liver index; (D–J) Serum levels of AST, ALT, ALP, GGT, TBA, TBIL, and DBIL in mice subjected to different treatments were determined using serology. For the statistical significance of this figure, bars indicate means ± SEM, *n* ═ 8 per group, ^###^*P* < 0.001 compared with the control, and ^*^P < 0.05, ^**^P < 0.01, ***P < 0.001 compared with the model. QE: Quercetin; AST: Aspartate aminotransferase; ALT: Alanine aminotransferase; ALP: Alkaline phosphatase; GGT: γ-glutamyl transferase; TBA: Total bile acid; TBIL: Total bilirubin; DBIL: Direct bilirubin.

### Ethical statement

Animal studies were performed in accordance with institutional guidelines approved by the Institutional Animal Care and Use Committee of Nanjing University of Chinese Medicine (Ethical number:16.05.2023/012072003037). Animal studies adhered to the recommendations outlined in the National Research Council’s Guide for the Care and Use of Laboratory Animals.

### Statistical analysis

Statistical analyses were conducted using GraphPad Prism 8.0 software. The results were presented as means ± standard error of the mean (SEM), indicating normal distribution and homogeneity of variance. Pairwise comparisons were performed using the *t*-test, while multigroup comparisons were completed through one-way analysis of variance. Post-tests were conducted using Tukey’s multiple comparison test. A two-sided test was used to obtain the *P* value, where *P* < 0.05 was considered statistically significant. Experiments were repeated at least three times and each condition was independently analyzed.

## Results

### QE alleviates liver injury in CCl4-induced mice

The use of repeated CCl4 administration is a well-established model for inducing liver fibrosis in vivo [30]. We implemented this model in mice to assess the liver-protective effects of QE (Figure 1A). At the end of the study, we calculated the body weight and liver index. Our findings revealed that treatment with colchicine and various concentrations of QE reduced the liver index, which had been increased by CCl4, without significantly affecting body weight (*P* < 0.05, Figure 1B and 1C). Serum markers of liver injury, including ALT, AST, ALP, GGT, TBA, TBIL, and DBIL, were all significantly elevated in the model group. However, treatment with colchicine and various concentrations of QE effectively reversed these elevated levels induced by CCl4, with the most notable effects observed in the H-QE group (*P* < 0.01, Figure 1D–1J). These results suggest that QE has the potential to alleviate liver injury and improve liver function in cases of CCl4-induced damage.

### QE alleviates liver fibrosis in CCl4-treated mice

**Figure 2.**
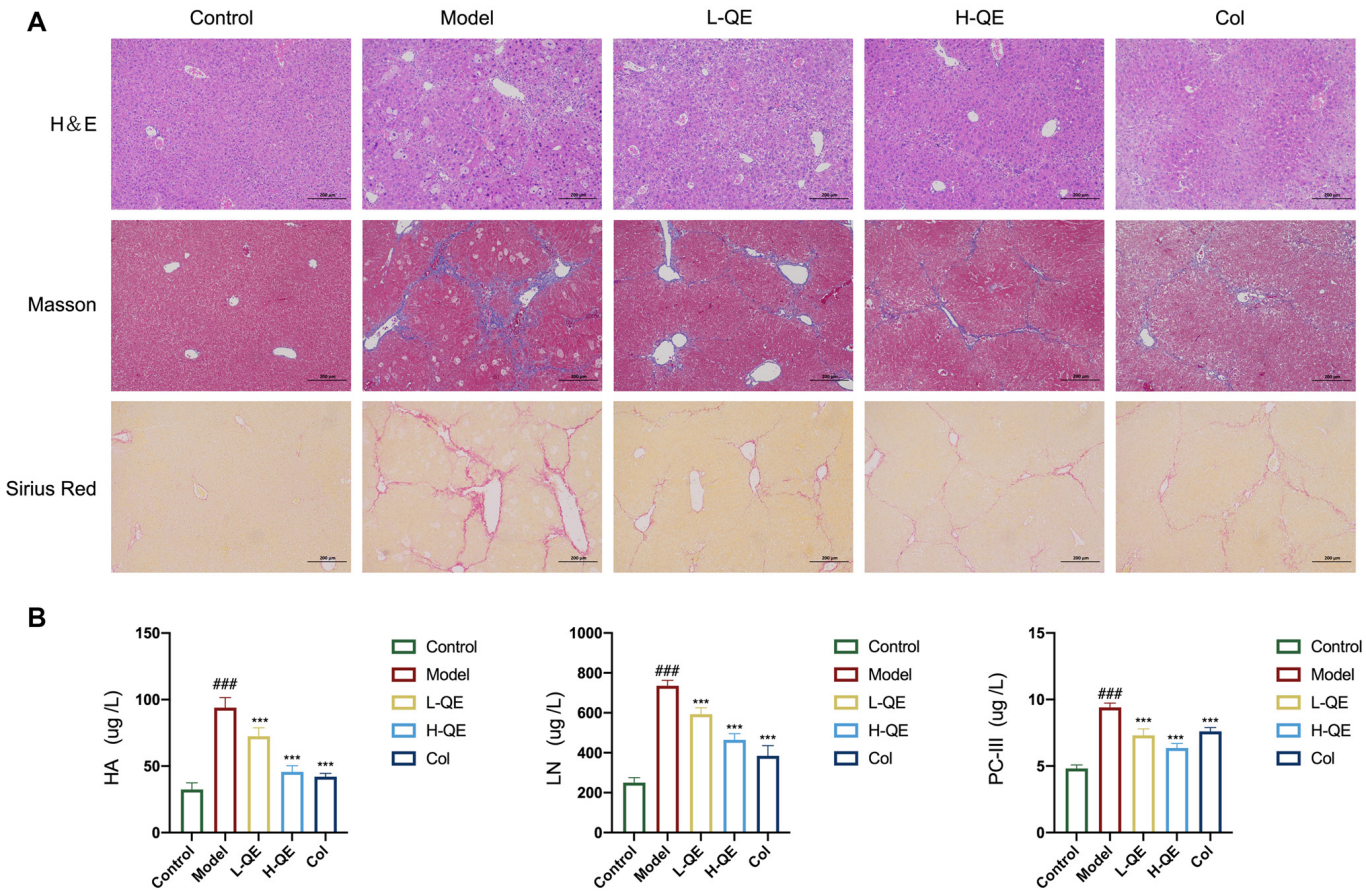
**Efficacy of QE in the treatment of liver fibrosis.** (A) H&E, Masson and Sirius red staining in liver tissues, scale bar: 200 µm; (B) Serum levels of HA, LN, and PC-III in mice subjected to different treatments were determined using serology. For the statistical significance of this figure, bars indicate means ± SEM, and *n* ≥ 3 per group, ^###^*P* < 0.001 compared with the control, and *P < 0.05, **P < 0.01, ***P <0.001 compared with the model. QE: Quercetin; HA: Hyaluronic acid; LN: Laminin; H&E: Hematoxylin and eosin; SEM: Standard error of the mean.

To further investigate the impact of QE on liver fibrosis, we conducted a thorough analysis of histology and collagen deposition in liver sections using H&E, Masson, and Sirius red staining (Figure 2A). H&E staining revealed disorganization of the liver structure and extensive inflammatory infiltration in the model mice group. Notably, treatment with colchicine and various concentrations of QE, particularly in the H-QE group, significantly mitigated these pathological changes and improved liver tissue structure. Analysis of the liver tissues using Masson and Sirius red stains indicated pronounced collagen deposition in the model group. However, treatment with QE or colchicine resulted in a significant decrease in collagen fiber accumulation. Moreover, we examined the serum levels of HA, LN, and PC-III, which are markers of liver fibrosis, and observed a significant increase in the model group, followed by a decrease after treatment with QE or colchicine (*P* < 0.01, Figure 2B). These results suggest that QE has a protective effect on liver fibrosis.

**Figure 3.**
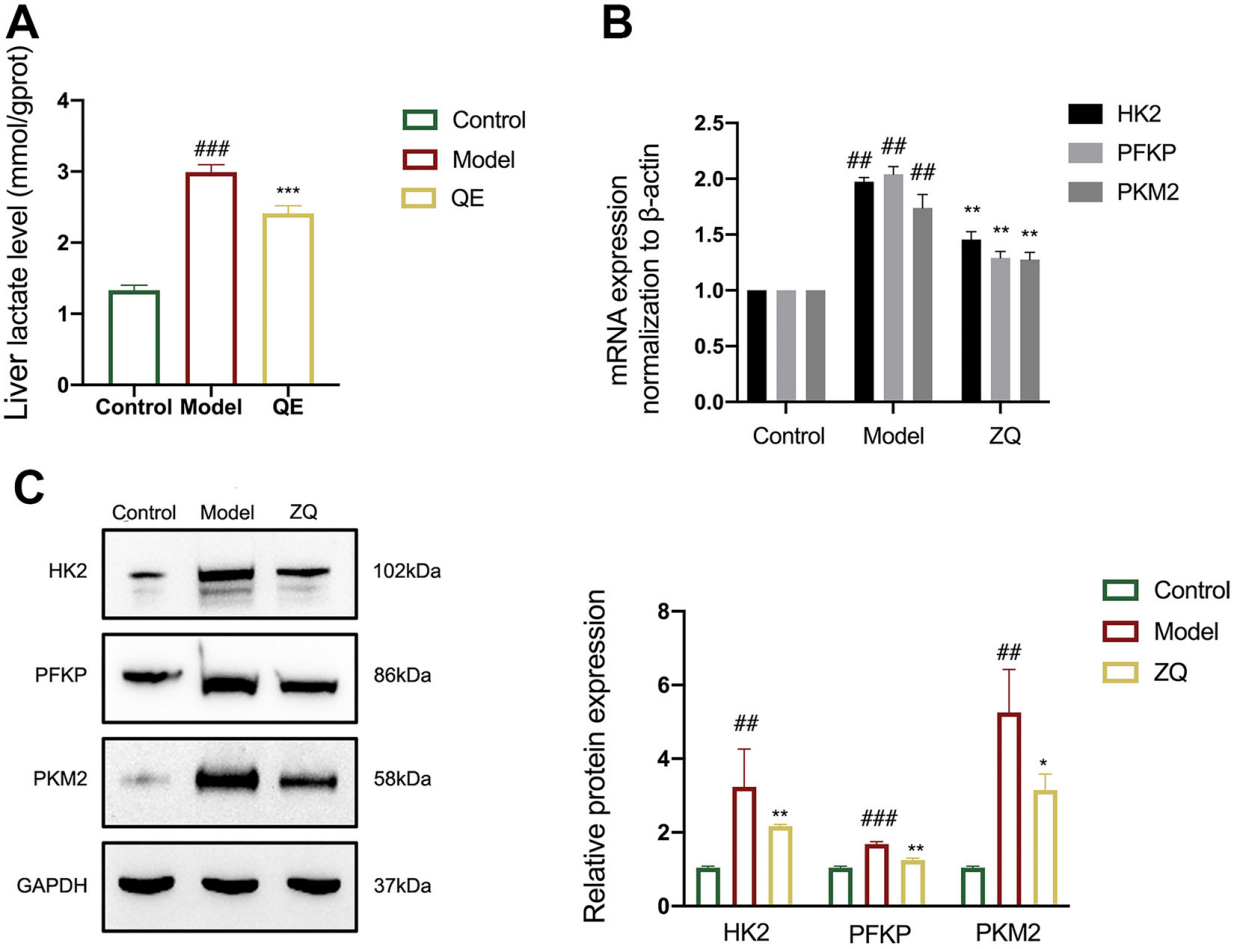
**QE inhibits glycolysis of CCl4-induced liver fibrosis in mice**. (A) Lactic acid levels in mice subjected to different treatments were detected using serological analysis; (B) The RT-qPCR results showed each group’s mRNA expressions of HK2, PFKP, and PKM2 in the liver tissues. β-Actin was used as the loading control; (C) Western blot analysis revealed the protein expressions of HK2, PFKP, and PKM2 in liver tissues from each group. GAPDH was used as the loading control. For the statistical significance of this figure, bars indicate means ± SEM, and *n* ≥ 3 per group, ^###^*P* < 0.001 compared with the control, and *P < 0.05, **P < 0.01, ***P < 0.001 compared with the model. QE: Quercetin; PKM2: Pyruvate kinase M2; PFKP: Phosphofructokinase platelet; GAPDH: Glyceraldehyde-3-phosphate dehydrogenase; RT-qPCR: Quantitative real-time PCR.

**Figure 4.**
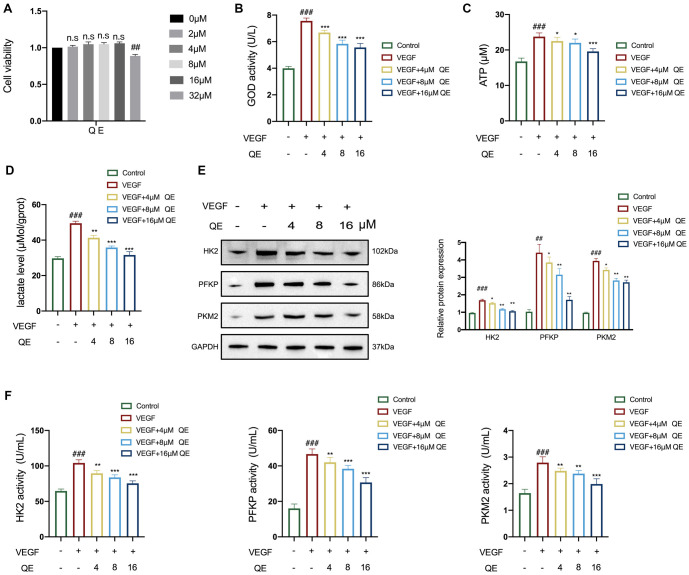
**Inhibition of QE on LSEC glycolysis.** (A) Effect of QE on cell viability was detected using the CCK8 reagent; (B) Measurements of glucose consumption are indicated by GOD activity; (C) Measurements of ATP levels; (D) Measurements of intracellular lactate levels; (E) Western blot analyses of PKM2, PFKP, and HK2 protein expression with quantification; (F) Measurements of intracellular enzyme activities of PKM2, PFKP, and HK2. For the statistical significance of this figure, bars indicate means ± SEM, and *n* ≥ 3 per group, ^###^*P* < 0.001 compared with the control, and *P < 0.05, **P < 0.01, ***P < 0.001 compared with the model. QE: Quercetin; PKM2: Pyruvate kinase M2; PFKP: Phosphofructokinase, platelet; LSEC: Liver sinusoidal endothelial cell; CCK8: Cell counting kit 8; GOD: Glucose oxidase; ATP: Adenosine triphosphate; HK2: Hexokinase II; SEM: Standard error of the mean.

**Figure 5.**
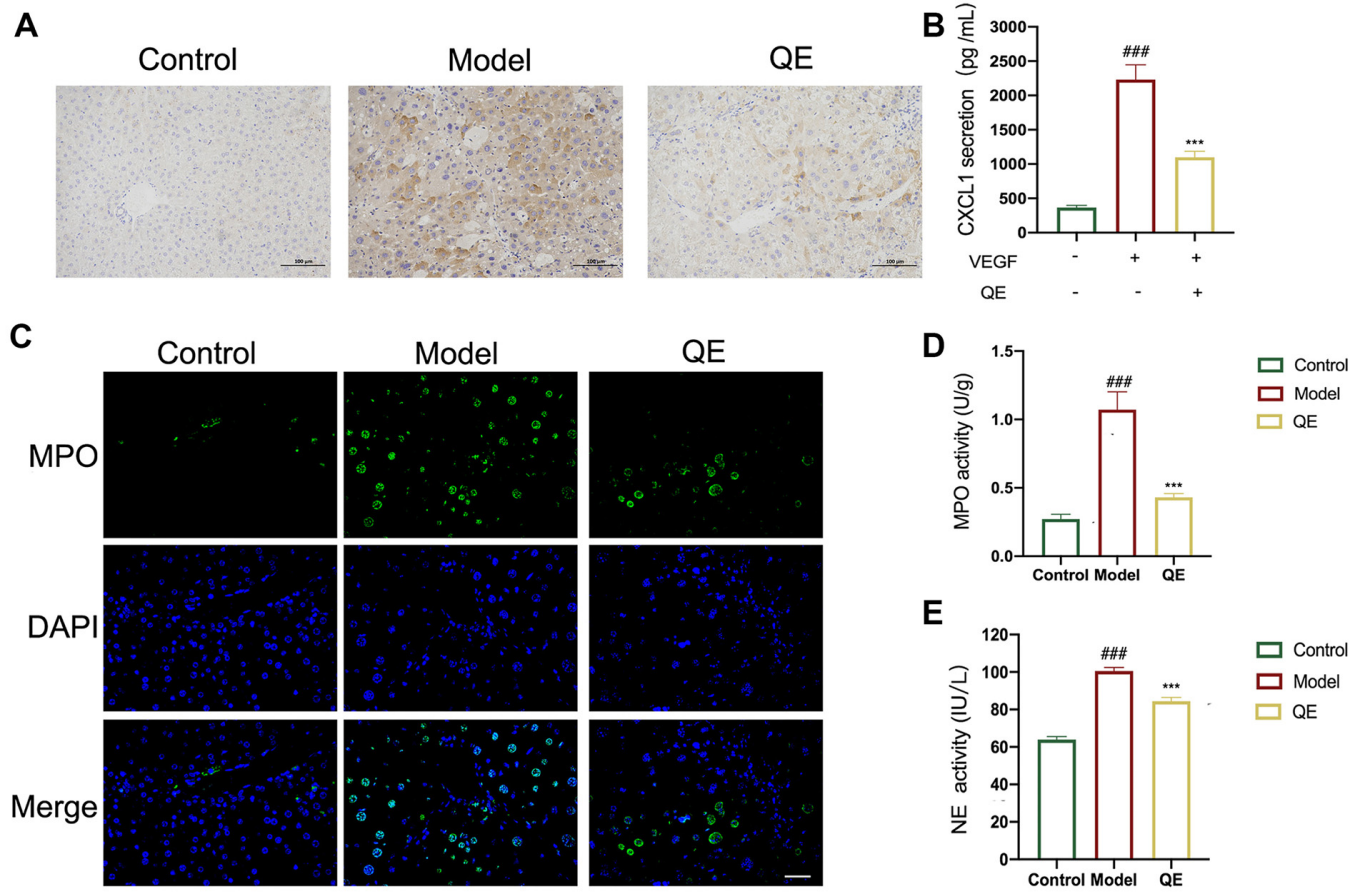
**Effect of QE on the neutrophil infiltration.** (A) Immunohistochemistry analysis of CXCL1 in liver tissues. Scale bars: 100 µm; (B) The level of CXCL1 release in the culture medium was evaluated using an ELISA kit; (C) Immunofluorescence analysis of MPO in liver tissues. Cells were stained for DAPI (blue) and MPO (green). Scale bars: 100 µm; (D) The expression levels of MPO in liver tissue; (E) The expression levels of NE in liver tissue. For the statistical significance of this figure, bars indicate means ± SEM, and *n* ≥ 3 per group, ^###^ *P* < 0.001 compared with the control, and *P < 0.05, **P < 0.01, ***P < 0.001 compared with the model. QE: Quercetin; ELISA: Enzyme-linked immunosorbent assay; MPO: Myeloperoxidase; DAPI: Diamidino-2-phenylindole; IHC: Immunohistochemistry; NE: Neutrophil elastase; SEM: Standard error of the mean.

**Figure 6.**
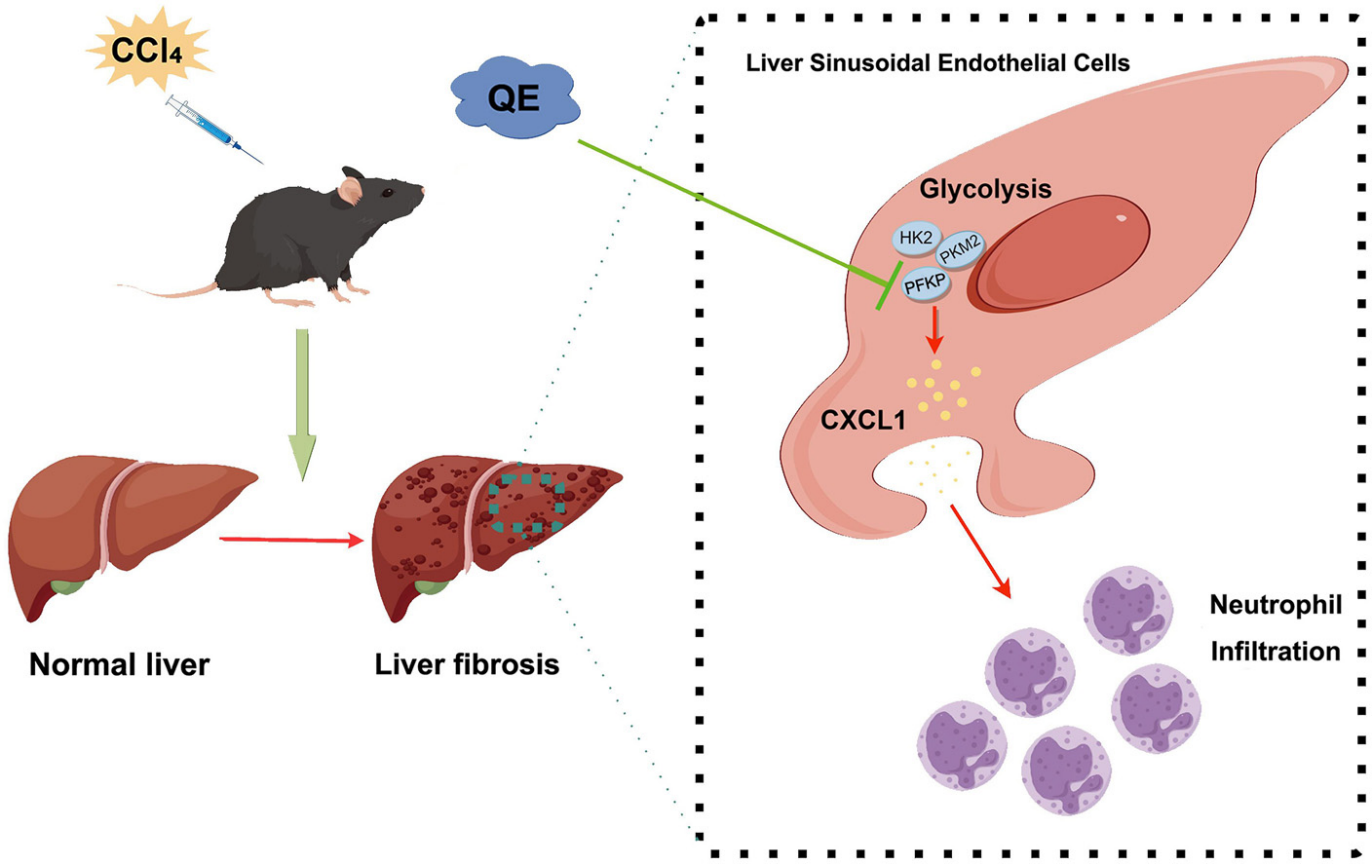
**Diagram of QE alleviating liver fibrosis via LSEC glycolysis and neutrophil infiltration.** QE: Quercetin; LSEC: Liver sinusoidal endothelial cell; HK2: Hexokinase II; PFKP: Phosphofructokinase platelet; PKM2: Pyruvate kinase M2.

### QE regulates glycolysis of CCl4-induced liver fibrosis in mice

Glycolysis, the sequential breakdown of glucose into pyruvate and lactate via enzymatic reactions, is a critical process involved in liver fibrosis. To investigate the impact of QE treatment on glycolysis, mice with CCl4-induced liver fibrosis were evaluated. Colorimetric analysis determined higher levels of lactate in the livers of mice in the model group compared to the control group. However, treatment with QE resulted in a significant decrease in liver lactate levels (*P* < 0.05, Figure 3A). Furthermore, RT-qPCR and western blotting analyses were carried out to assess the mRNA and protein expressions of glycolytic enzymes HK2, PFKP, and PKM2 in vivo. These analyses revealed a substantial increase in the expression of these enzymes in the model group, with a significant reduction following QE treatment (*P* < 0.01, Figure 3B and 3C). Collectively, these findings demonstrate the ability of QE to regulate glycolysis in liver fibrosis.

### QE attenuates the glycolysis in LSEC

Endothelial cells are highly glycolytic [28], and targeting LSEC glycolysis might be a therapeutic approach for reversing liver fibrosis. Therefore, we investigated the regulatory mechanisms of QE in LSEC glycolysis. We first identified the effects of different QE concentrations on the viability of LSECs using the CCK8 assay (Figure 4A). Cell viability significantly decreased when the QE concentration reached 32 µM. Therefore, we selected concentrations of 4, 8, and 16 µM for subsequent experiments. To mimic the growth environment in a fibrotic liver, we used 40 ng/mL of VEGF to stimulate LSECs, followed by QE treatment. The results showed that QE reduced glucose consumption in LSECs in a concentration-dependent manner, as evidenced by the changes in GOD activity (*P* < 0.05, Figure 4B). Simultaneously, intracellular lactic acid and ATP levels were reduced by QE in a concentration-dependent manner in LSECs (*P* < 0.01, Figure 4C and 4D). Furthermore, the effects of QE on glycolytic enzyme activities and protein expression levels of HK2, PFKP, and PKM2 were examined. We found that the intracellular activities of these glycolytic enzymes were distinctly decreased by QE (*P* < 0.01, Figure 4E). Meanwhile, their protein expressions were downregulated by QE (*P* < 0.01, Figure 4F) in the LSECs as well. These observations demonstrated effective diminution of glycolysis in the LSECs upon QE treatment.

### QE attenuates the neutrophil infiltration

Previous studies have shown that inhibiting glycolysis, specifically in endothelial cells (ECs), can lower CXCL1 production and is closely linked to the infiltration of neutrophils in liver fibrosis [31]. Therefore, further investigations are required to determine if QE can alleviate liver fibrosis by reducing CXCL1 production by inhibiting EC glycolysis and limiting neutrophil infiltration. Initially, we examined the impact of QE on the expression of CXCL1, a chemokine that attracts neutrophils. IHC demonstrated elevated levels of CXCL1 in the livers of mice in the model group compared to those in the control group. In contrast, treatment with QE decreased the levels of CXCL1 in liver tissue (Figure 5A). Similarly, in vitro experiments showed a decrease in CXCL1 levels in the culture medium with QE treatment (*P* < 0.01, Figure 5B). To further investigate the effect of QE on neutrophil infiltration, we analyzed the levels of MPO and NE, known markers of neutrophil activation and infiltration [32, 33]. MPO IF staining revealed significantly lower levels of MPO in the QE group compared to the model group (Figure 5C). This finding was verified through MPO activity assays (*P* < 0.01, Figure 5D). In addition, we observed a considerable reduction in NE activity in liver tissues following QE treatment (*P* < 0.01, Figure 5E). These observations suggest that QE effectively reduces CXCL1 secretion and neutrophil infiltration during the process of liver fibrosis.

## Discussion

Liver fibrosis is the pivotal pathological stage of chronic liver disease, and active and effective intervention strategies can considerably affect the transformation of liver fibrosis to cirrhosis and hepatocellular carcinoma [5, 34]. Through our research, we have proven the effectiveness of QE in mitigating liver injury and fibrosis caused by CCl4 in a mouse model (Figures 1 and 2).

Cellular metabolism plays a crucial role in fibrogenic response. Research shows that diseased livers accumulate glycolytic stromal cells, correlating with liver fibrosis severity [35]. Therefore, our research focused on exploring QE-induced alterations in hepatic glycolysis during liver fibrosis. The experimental findings indicated that QE effectively downregulates the expression of glycolytic products and enzymes (Figure 3), suggesting that QE’s therapeutic effects may stem from its ability to regulate aberrant hepatic glycolysis.

LSECs are situated at the critical interface of material exchange between hepatic sinusoidal blood flow and hepatic parenchymal cells [36], playing a pivotal role in maintaining liver homeostasis and regulating the quiescent state of stellate cells and Kupffer cells [37]. However, LSECs are highly susceptible to changes in the microenvironment, and prolonged liver injury can potentially induce aberrant activation of LSECs, leading to inflammation and fibrosis [7]. Recent research revealed that LSECs (the predominant endothelial cell population within the hepatic milieu) primarily rely on glycolysis for ATP production to support their functionality, in contrast to oxidative phosphorylation [38]. In summary, LSECs are crucial in the development of liver fibrosis by regulating glycolytic metabolism. Our experimental findings further confirmed this by revealing a significant increase in glycolysis activity within LSECs. Simultaneously, QE treatment effectively regulated intracellular glucose consumption and ATP and lactate levels in the LSECs and decreased the expression and activity of glycolytolytic enzymes, including HK2, PFKP, and PKM2 (Figure 4). This prompted a shift in our research focus to the therapeutic implications of QE on the glycolytic processes within LSECs.

Recent studies revealed the pivotal role of the glycolytic activity of LSECs in liver fibrosis via the regulation of neutrophil infiltration [12]. The different CXCL chemokines secreted by LSECs, particularly CXCL1, are central to this process. CXCL1 effectively enhances neutrophil aggregation and triggers the formation of neutrophil infiltration, thus increasing portal vein pressure and fibrosis. Consequently, inhibiting CXCL1 release induced by LSEC glycolysis is paramount for intervening in liver fibrosis [11]. Our in vivo and in vitro experiments provided compelling evidence for the ability of QE to inhibit CXCL1 secretion (Figure 5A and 5B). These findings reinforced our confidence in the potential of QE to mitigate CXCL1-associated neutrophil infiltration. Furthermore, our conjecture is supported by the MPO and NE experiments (Figure 5C and 5D). QE could significantly reduce the levels of MPO and NE, further verifying the effectiveness of QE in inhibiting neutrophil infiltration in liver fibrosis.

## Conclusion

In conclusion, this study elucidated the protective mechanisms of QE against liver fibrosis. The influence of QE was exerted through the inhibition of glycolytic activities within LSECs, mitigating neutrophil infiltration and the subsequent amelioration of liver injury and fibrosis induced by CCl4. These findings offer novel insights into the molecular mechanisms of QE in the prevention and treatment of liver fibrosis (Figure 6).

## Data Availability

The datasets generated and/or analyzed during the current study are available from the corresponding author (F.Y.) on a reasonable request.
